# Muscular Versus Non-Muscular Free Flaps for Soft Tissue Coverage of Chronic Tibial Osteomyelitis

**DOI:** 10.29252/wjps.7.3.294

**Published:** 2018-09

**Authors:** Pablo Buono, Pascal Castus, Victor Dubois-Ferrière, Eva Meia Rüegg, Ilker Uçkay, Mathieu Assal, Brigitte Pittet-Cuénod, Ali Modarressi

**Affiliations:** 1 Department of Plastic, Reconstructive and Aesthetic Surgery, Geneva University Hospitals, Faculty of Medicine, University of Geneva, Switzerland;; 2 Orthopaedic Surgery Department, Geneva University Hospitals, Faculty of Medicine, University of Geneva, Switzerland;; 3 Unit of Infectious Diseases, Geneva University Hospitals, Faculty of Medicine, University of Geneva, Switzerland

**Keywords:** Lower limb, Chronic osteomyelitis, Free flap, Reconstructive surgery, Microsurgery

## Abstract

**BACKGROUND:**

Eradication of chronic tibial osteomyelitis necessitates aggressive debridement is often followed by soft tissue reconstruction. Muscular flaps are said to be more effective than non-muscular flaps for infection treatment, while fasciocutaneous and perforator flaps are considered to be less invasive and offering a better aesthetic result.

**METHODS:**

In this study, we reviewed 24 consecutive cases of chronic tibial osteomyelitis treated according to a specific protocol in a tertiary hospital. Soft tissue coverage was done with either muscular or non-muscular free flaps. Infection recurrence and complications were compared between different flap types. Additionally, we assessed the long-term functional and aesthetic results and patient’s satisfaction.

**RESULTS:**

Muscular flap was used in 13 patients (13 latissimus dorsi and 1 serratus anterior) and 11 patients underwent fasciocutaneous/perforator flaps [1 anterolateral thigh flap, 4 lateral arm flaps, 5 thoracodorsal artery perforator (TAP) flaps and 1 radial forearm flap]. Infection was resolved for 84.6% of patients in the muscular flaps group and 90.9% in the non-muscular flaps group. None of the patients with muscular flaps were satisfied with the aesthetic appearance of their reconstructed leg when compared to 83.3% of patients with non-muscular flaps. Also, a slight regain of touch sensitivity was acknowledged in the non-muscular flap group compared to the muscular.

**CONCLUSION:**

In this study of adult chronic tibial osteomyelitis cases, we demonstrated that fasciocutaneous and perforator free flaps offer a comparable efficacy to the muscle flaps for infection treatment, with a significantly higher patient satisfaction and aesthetic result.

## INTRODUCTION

Successful treatment of chronic osteomyelitis of the adult tibia is cumbersome and expensive.^1^ Bone sequesters and defects, poorly vascularized scar tissue and fragile soft tissue surroundings are major local obstacles to recovery. Moreover, patients often present substantial comorbidities and a history of multiple previous operations that render treatment complex.^2^ First publications targeting limb preservation for chronic tibial osteomyelitis cases reported a recurrence risk of 30%.^3^ Nowadays, a more aggressive surgical approach has shown to improve long-term remission with a recurrence of less than 5% ^2^^-^^5^

Four key principles for optimal management of chronic osteomyelitis are established: (i) complete debridement of devascularized bone and soft tissue, (ii) adequate stabilization of the bone, (iii) targeted antibiotic therapy, and (iv) coverage with well vascularized soft tissue.^6^^,^^7^ Controversies still exist concerning the type of flaps for soft tissue reconstruction. Number of studies have pointed out the muscle as an adequate coverage in the context of infection,^6^^-^^14^ that was proposed and reinforced by the early works of others,^6^^,^^7^^-^^13^ as well as several animal experiments.^15^^-^^18^

In contrast, more recent publications identified a superior efficacy of fascio-cutaneous and, more lately, perforator flaps, over muscle/musculo-cutaneous flaps in lower acute limb reconstructions after trauma.^1^^,^^19^^-^^26^ Few studies have been published concerning the potential use of these non-muscular flaps in the particular case of chronic inferior limb osteomyelitis^18^^,^^24^^,^^27^ and there is a lack of studies comparing the efficacy of non-muscular to muscular for chronic osteomyelitis treatment. The aim of this study is to compare two sets of patients treated for chronic osteomyelitis of the tibia in which the management differs only in the type of the flap (i.e. non muscular, muscular) that was used for the soft tissue reconstruction.

## MATERIALS AND METHODS

We reviewed medical files of 24 consecutive patients treated for chronic osteomyelitis of the tibia in our institution over 13 years. Inclusion criteria were: (a) chronic tibial osteomyelitis of at least 3 months duration; (b) clinical signs of chronic osteomyelitis (i.e. pain, fistulas with purulent drainage, compromised bone coverage by the soft tissues); (c) radiographic findings consistent with chronic bone infection (i.e. sequestrum, involucrum, lytic lesions); (d) positive bacterial cultures in bone specimens; (e) >1 year follow-up post-flap surgery and (f) patients older than 16 years. 

All patients were treated by a multidisciplinary team, including orthopaedic surgeons, infectious diseases specialists, physiotherapists and plastic surgeons. All patients received the same 2-step-treatment protocol: the first step involved a thorough debridement of necrotic bone, scar tissue and metalwork if present, associated with the implantation of gentamicin polymethyl metacrylate beads into the bone defect. In case of bone instability, we applied an external fixation. 

All wounds were left open and dressing changes were performed 3 to 5 times a week for wound assessment and sampling. The infectious diseases specialist adapted the antimicrobial agents to the bacteriological species and susceptibility testing and administered them for 12 weeks. The second stage occurred in the second to third week, with removal of the gentamicin beads and implementation of bone grafts. Soft tissue coverage was performed by plastic surgeons using a free flap. The flap type was chosen according to defect size, arteriographical results and patients’ preferences. An internal fixation was performed in case of instability. 

According to medical files review, results were expressed considering immediate post-operative evolution, complications and re-operations and the presence or absence of clinical and radiological signs of recurrent osteomyelitis at the last follow-up. Results were compared between patients who underwent a muscular free flap procedure (M group) to those with non-muscular flap (non-M group). Moreover, for this study, all patients were re-invited for clinical evaluation. During this consultation, we assessed signs of osteomyelitis recurrence, quality of the soft tissue coverage, and aesthetic outcome (simple questionnaire). Both physician (not the operator) and the patient were evaluated by a questionnaire, the tissue thickness, texture and aesthetic appearance (e.g. relief, color match, symmetry with healthy side). Neurologic sensory testing was performed by pinprick, skin temperature, two-point-discrimination and light touch tests. 

## RESULTS

The average age of the study population was 41 years (range: 16 to 75 years); while 18 patients were males. All 24 patients presented a Cierny grade 3 to 4 of chronic osteomyelitis. All patients, except one, revealed chronic osteomyelitis following osteosynthesis of an open fracture of their tibia. The remaining patient developed a surgical site infection after valgus osteotomy. The delay between the initial trauma and the treatment ranged from 3 months to 22 years, averaging 7.5 years. The average soft tissue defect after debridement (stage 1) was 87.89 cm^2^ (18 to 225 cm^2^). 

CT-angiogram or invasive arteriography identified 12 patients with good patency of all 3 limb vessels (7 in the M group and 5 in the non-M group). The remaining 12 patients had severe to complete stenosis of at least one vessel: complete occlusion of 1/3 vessel was found in 2 patients in the M group and 5 in the non-M group. Complete occlusion of 2/3 vessels was found in 3 members of the M group in contrast to 0 in the non-M group. We performed 25 flaps: 14 muscular flaps (M group), including 8 musculocutaneous flaps (all latissimus dorsi) and 6 muscle flaps covered by a split-thickness skin grafts (5 latissimus dorsi and 1 serratus anterior). 

**Fig. 1:**
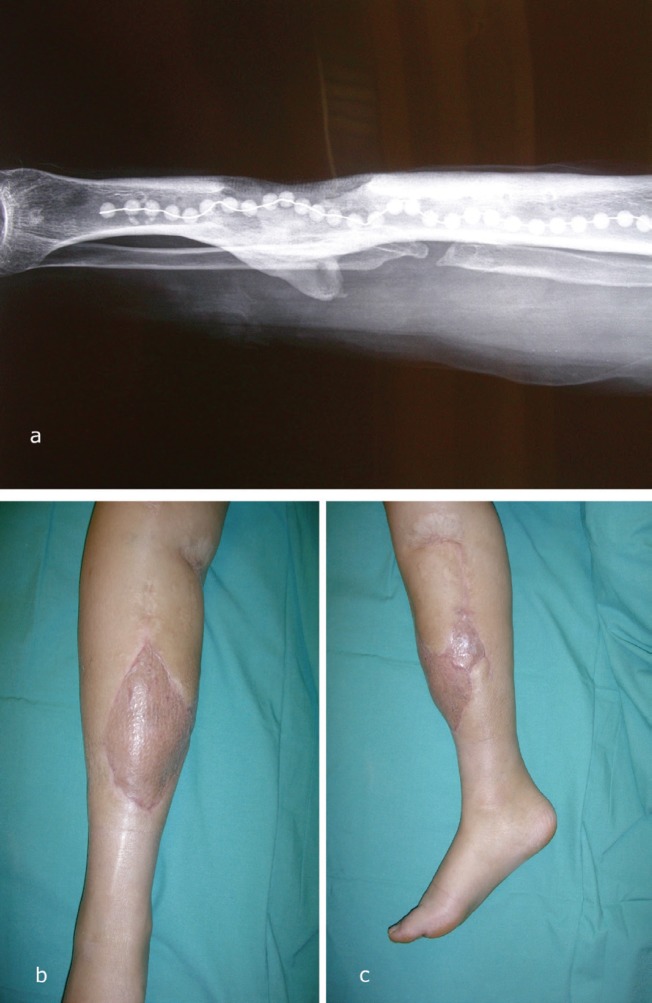
Case example from M-group: muscular latissimus dorsi flap with split-thickness skin graft. Radiologic images showed a partial diaphysal defect of tibia and a strong cortical thickening due to the chronic inflammation (Figure 1a). A free muscular latissimus dorsi flap covered by a split-thickness skin-graft, anastomosed to the posterior tibial vessels was used for coverage of the defect (Figure 1b and 1c)

Figure 1 is from M-group who was a 34-year-old female patient, victim of an accident with a landmine nine years before. The left leg was amputated at mid-leg and she was able to walk with prosthesis. She suffered from a chronic osteomyelitis with a osteocutaneous fistula from the tibia, infected with methicillin-resistant *Staphylococcus aureus* (MRSA) for many years. She presented an osteocutaneous defect of the anterior side of the right anterior lower leg after repeated surgical debridement and placement of gentamicine beads associated to an antibiotherapy by vancomycine. Radiologic images showed a partial diaphysal defect of tibia and a strong cortical thickening due to the chronic inflammation. A free muscular latissimus dorsi flap covered by a split-thickness skin-graft, anastomosed to the posterior tibial vessels was used for coverage of the defect.

**Fig. 2:**
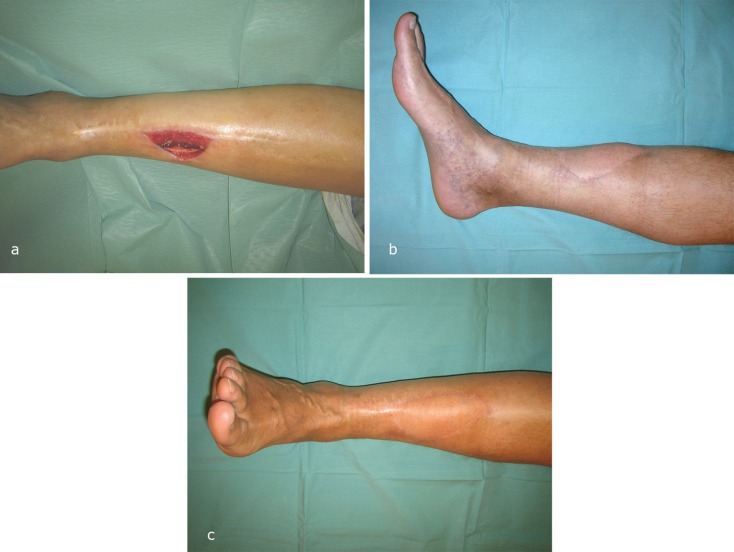
Case example from non-M-group: fasciocutaneous lateral arm flap. A patient presenting an osteocutaneous defect of the right anterior lower leg after repeated surgical debridement (Figure 2a). Clinical status at 1-year follow-up (Figure 2b and 2c) shows an excellent integration of the flap into the adjacent soft tissue

The 11 fasciocutaneous/perforator flaps (non-M group) were 1 anterolateral thigh flap, 4 lateral arm flaps (Figure 2), 5 thoracodorsal artery perforator (TAP) flaps and 1 radial forearm flap. There were 3 immediate flap failures (2 musculocutaneous latissimus dorsi and 1 TAP flap) due to vascular thrombosis. All failures occurred in cases where lengthening of the pedicle was required with a venous graft. Two cases were successfully re-operated, one with a second muscle latissimus dorsi muscle free flap and another with a cross leg flap. The third patient (from the M-group) underwent leg amputation due to very poor vascular status up to above the knee. 

At an average follow-up of 2.5 years (range, 1 to 12 y), 21 patients (87.5%) remained in remission; 11 from the M-group (84.6%) and 10 from non-M group (90.9%) (Table 1). Osteomyelitis recurred in 2 patients: one with muscular flap at 1 year, the other recurred in the non-M group after 2 years. The first patient was a severe active smoker and drug addict with borderline personality disorder. The second had the worst stenosis of all 3 limb vessels among the study population. Both ended with amputation. 

**Table 1:** Patients’ data from both muscular and non-muscular flap groups

**Variable**	**Muscular flap group** **(n=13)**	**Non-muscular flap group ** **(n=11)**
Gender (male/female)	9/4	9/2
Age mean (range)[years]	47.6 (24-75)	38.3 (16-69)
Active smokers	4	4
Diabetes mellitus	3	2
Cardiovascular disease	3	2
Obesity	3	1
Leg vascular status: 0 artery occlusion 1 artery occlusion >1 arteriers occlusion	7 3 3	5 5 1
Amputation	2	1
Flap failure	2	1
Infection healing	84.6%	90.9%
Follow-up mean (range) [years]	3.4 (1-12)	2.3 (1-4)

Figure 2 is from non-M-group who is a 60-year-old male patient presenting an osteocutaneous defect of the right anterior lower leg after repeated surgical debridement. Twenty-eight years ago, he had a mid-diaphyseal fracture of the right tibia treated by osteosynthesis. Seven years later, he developed a stress fracture and a chronic osteomyelitis with formation of a sequestrum and cutaneus fistulas. After nine years, the osteomyelitis was treated in a 2-step procedure: (i) the removal of the sequestrum and the temporary placement of gentamicine beads, and (ii) soft tissue coverage three weeks later with a free fasciocutaneous lateral arm flap anastomosed to the anterior tibial vessels. Clinical status at 1-year follow-up showed an excellent integration of the flap into the adjacent soft tissue. 

Among all patients, ten patients returned for clinical review: 4 from the M-group and 6 from the non-M group (Figure 1 and 2). All these 10 patients were infection-free. They had all achieved full weight bearing without pain and had returned to their previous level of activity. Regarding the aesthetic satisfaction, none of the four patients in the M-group was satisfied with the final appearance, whereas 5 of 6 patients in the non-M group described their leg as aesthetically acceptable (Fisher-exact-test, *p*=0.048). Two-point-discrimination, temperature and pain were impaired equally in both subgroups, only the “fine sensitivity” was different in favor of the non-M flaps (Table 2).

**Table 2:** Clinical examination of 10 patients form both muscular and non-muscular flap groups

**Variable**	**Muscular flap group** **(n=4)**	**Non-muscular flap group ** **(n=6)**
Gender (male/female)	2/2	6/0
Mean follow-up [years]	9.5	3
Bump	1	0
Depression	2	0
Color match :		
Good Darker Lighter	2 2 0	3 2 1
Flap consistency :		
Soft Indurated	3 1	6 0
Flap sensitivity :		
Pressure 2 points discrimination Temperature Pinprick test	0 0 0 0	>4.56g 0 0 0
Flap aesthetic satisfaction	0	5
Donor site pain/discomfort/disability	1	2
Donor site aesthetic satisfaction	2	5

## DISCUSSION

The works of Mathes *et al.*^8^^,^^9^ and others^6^^,^^7^^,^^10^^-^^13^ strongly influenced free-flap surgery, resuming that the muscle is the best coverage after debridement of infected tissue.^15^^-^^18^ Later on, however, the benefits of transferring fascio-cutaneous flaps without muscular component gained momentum for panoply of reasons: decreased donor site morbidity, better integration via skin-to-skin connections with the recipient site,^26^^,^^28^ better color matching, better contour compared to muscle and/or musculocutaneous flaps;^29^ easier debulking,^29^ and better neurologic re-sensitizing of the flap.^26^^,^^28^

Our study of adult chronic tibial osteomyelitis cases showed that fasciocutaneous and perforator free flaps, compared to the muscle flaps, yielded equal success in terms of infection recurrence. Interestingly patient’s satisfaction and aesthetic results were higher for those who underwent a non-muscular free flap reconstruction. Regarding sensibility, we lacked any positive results when testing with monofilaments in the muscle group, whereas all patients in the non-muscle group showed some positive stimuli to the test but for the highest levels of pressure. As the number of patients included in our study was low, particularly those being available for clinical exams, further studies are needed to confirm these results.

Given the advantages of fascio-cutaneous or perforator flaps, the use of non-muscular free flaps is currently our first-line option for chronic tibial osteomyelitis treatment in adults. In addition to the choice of the adequate flap, the debridement and infection treatment are key elements for the success of chronic osteomyelitis treatment. This complex treatment requires a multidisciplinary approach including orthopedists, plastic surgeons and infectious diseases specialists. 

## CONFLICT OF INTEREST

None declared.
